# The Relationship Between Medial Gastrocnemius Lengthening Properties and Stretch Reflexes in Cerebral Palsy

**DOI:** 10.3389/fped.2018.00259

**Published:** 2018-10-04

**Authors:** Lynn Bar-On, Barbara M. Kalkman, Francesco Cenni, Simon-Henri Schless, Guy Molenaers, Constantinos N. Maganaris, Alfie Bass, Gill Holmes, Gabor J. Barton, Thomas D. O'Brien, Kaat Desloovere

**Affiliations:** ^1^Department of Rehabilitation Sciences, KU Leuven, Leuven, Belgium; ^2^Amsterdam UMC, Vrije Universiteit Amsterdam, Department of Rehabilitation Medicine, Amsterdam Movement Sciences, Amsterdam, Netherlands; ^3^Research Institute for Sport and Exercise Sciences, Liverpool John Moores University, Liverpool, United Kingdom; ^4^Department of Mechanical Engineering, KU Leuven, Leuven, Belgium; ^5^Alder Hey Children's NHS Foundation Trust, Liverpool, United Kingdom

**Keywords:** cerebral palsy, stretch reflexes, spasticity, muscle stiffness, dynamic ultrasound, EMG, medial gastrocnemius

## Abstract

Stretch reflex hyperactivity in the gastrocnemius of children with spastic cerebral palsy (CP) is commonly evaluated by passively rotating the ankle joint into dorsiflexion at different velocities, such as applied in conventional clinical spasticity assessments. However, surface electromyography (sEMG) collected from the medial gastrocnemius (MG) during such examination reveals unexplained heterogeneity in muscle activation between patients. Recent literature also highlights altered muscle tensile behavior in children with spastic CP. We aimed to document MG muscle and tendon lengthening during passive ankle motion at slow and fast velocity and explore its interdependence with the elicited hyperactive stretch reflex. The ankle of 15 children with CP (11 ± 3 years, GMFCS 9I 6II, 8 bilateral, 7 unilateral) and 16 typically developing children (TDC) was passively rotated over its full range of motion at slow and fast velocity. Ultrasound, synchronized with motion-analysis, was used to track the movement of the MG muscle-tendon junction and extract the relative lengthening of muscle and tendon during joint rotation. Simultaneously, MG sEMG was measured. Outcome parameters included the angular and muscle lengthening velocities 30 ms before EMG onset and the gain in root mean square EMG during stretch, as a measure of stretch reflex activity. Compared to slow rotation, the muscle lengthened less and stretch reflex activity was higher during fast rotation. These velocity-induced changes were more marked in CP compared to TDC. In the CP group, muscle-lengthening velocity had higher correlation coefficients with stretch reflex hyperactivity than joint angular velocity. Muscles with greater relative muscle lengthening during slow rotation had earlier and stronger stretch reflexes during fast rotation. These initial results suggest that ankle angular velocity is not representative of MG muscle lengthening velocity and is less related to stretch reflex hyperactivity than MG muscle lengthening. In addition, muscles that lengthened more during slow joint rotation were more likely to show a velocity-dependent stretch reflex. This interdependence of muscle lengthening and stretch reflexes may be important to consider when administering treatment. However, muscle and tendon lengthening properties alone could not fully explain the variability in stretch reflexes, indicating that other factors should also be investigated.

## Introduction

Cerebral palsy (CP), the most common childhood disability, is an umbrella diagnosis attributed to a lesion in the developing fetal or infant brain (1). Depending on the timing, location, type and extent of the lesion, the clinical manifestation is highly variable. The different forms of CP are categorized by the prevalent tone abnormality (dystonia, ataxia, or spasticity) and by the distribution of the impairments (unilateral vs. bilateral). Spasticity, defined as velocity-dependent stretch reflex hyperactivity (2) is diagnosed in 80% of the cases and has been indicated to contribute to gait deviations including limited dorsiflexion during swing and at initial contact, decreased ankle dorsiflexion during mid-stance and limited push off power at terminal stance (3). Furthermore, it is long thought that stretch reflex hyperactivity contributes to progressively worsening secondary musculoskeletal impairments including increased muscle stiffness, contracture and eventually deformities of immature skeletal bones (4). Based on this assumption, early aggressive treatment of stretch reflex hyperactivity is commonly indicated to delay and reduce the need for orthopedic surgery (5). Proper diagnosis of stretch reflex hyperactivity is therefore clinically relevant and a deeper understanding of its relationship with muscle dynamics is currently lacking.

Stretch reflex hyperactivity in the gastrocnemius of children with spastic CP is commonly evaluated by passively rotating the ankle joint into dorsiflexion at different velocities, such as applied in conventional clinical spasticity assessments (6). However, latest research using surface electromyography (sEMG) from the medial gastrocnemius (MG) during such examination reveals unexplained heterogeneity in muscle activation between patients (7). High variability was noted, both in the amplitude of the stretch reflex response as well as the threshold velocity at which muscle activation occurred during passive stretch, the so-called stretch reflex threshold (SRT). The reason why some muscles and some children have a lower SRT velocity is yet to be confirmed. One hypothesis is that the activation threshold is sensitive to muscle properties, such as its ability to lengthen when brought under stretch (8).

Using dynamic ultrasound (US) imaging to assess muscle lengthening, it has been shown that, in comparison to typically developed muscles, the MG muscle belly in children with spastic CP has a reduced lengthening ability during slow passive ankle rotation (9, 10). In addition, during the mid-stance phase of gait, muscle fascicles lengthen more in CP compared to typically developing children (11, 12). These findings suggest that, in both passive and active conditions, muscle-tendon interaction during joint rotation is altered. Such a redistribution of the movement between the tendon and muscle fascicles may increase the proportion of fascicle lengthening during passive or active movement, thus triggering more muscle receptors (i.e., spindles), increasing afferent activity, and consequently increasing the stretch reflex response (13).

Additionally, these particular alterations highlight that the muscle and tendon mutually modulate their behavior when the joint is rotated. Consequently, any assessment at the joint may not represent the properties and behavior of the muscle and tendon similarly across children. The wide inter-child variability observed in the SRT when expressed as a joint angular velocity (14) may therefore be explained by differences in muscle tensile properties that alter muscle behavior during stretch.

By combining dynamic US imaging with sEMG measurements during fast passive muscle stretch, we can now quantify the SRT in terms of muscle lengthening velocity instead of joint angular velocity. As such, the mechanisms that trigger stretch reflexes at the level of the muscle, where the stretch receptors are located, can be directly investigated to allow better understanding of the variability in SRT sensitivity among different muscles and children. Such an investigation may also help establish the relationship between primary neural symptoms and secondary musculoskeletal alterations. Deciphering the inter-dependence of impairments in CP can result in a shift of the treatment focus, particularly at an early stage. Furthermore, understanding the sources of variability in clinical symptoms may help to develop personalized treatment (15). This is particularly important since it is debatable whether stretch reflex hyperactivity plays a dominant role in impairing gait (16–18).

Therefore, the aim of this study was to explore the relationship between MG muscle and tendon lengthening and hyperactive stretch reflexes recorded during slow and fast passive ankle rotations in children with spastic CP and typically developing children (TDC). We hypothesized that analysis at the level of the muscle, rather than at the joint, will give a better understanding of the triggers of stretch reflex hyperactivity.

## Materials and methods

### Participants

Children diagnosed with spastic CP and TDC, aged between 6 and 16 years, were recruited for this multicentre study from the University Hospital of Leuven and from the Alder Hey Children's Hospital in Liverpool. Children with CP were excluded when diagnosed with ataxia, dystonia, severe plantar flexor muscle weakness [manual muscle test < 1+ (19)], bony deformities or contractures resulting in less than 20 degrees of ankle range of motion in the sagittal plane, cognitive problems that could impede the measurements, botulinum toxin-A injections 6 months prior to the measurement, an intrathecal baclofen pump, selective dorsal rhizotomy, or any previous orthopedic surgery below the knee. TDC had no history of orthopedic or neurological impairments. This study was carried out in accordance with the recommendations of the National Health Service, and by the University Hospital's ethics committee in Leuven, Belgium (study number s57384). The protocol was approved by the same bodies. Written parental consent was obtained, and written assent was given by children in accordance with local regulations and with the Declaration of Helsinki.

### Measurement protocol

**Figure 1:**
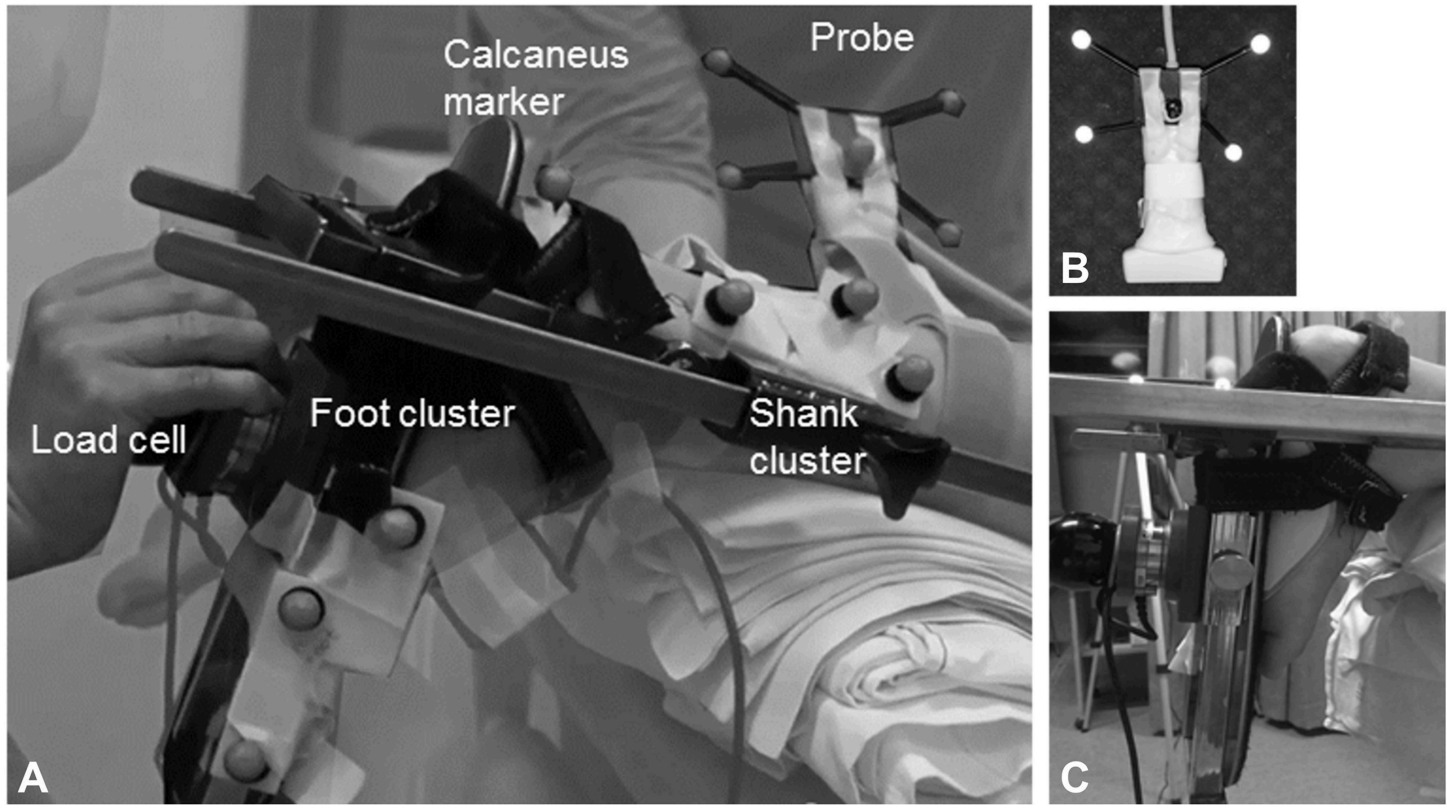
**(A)** Experimental design of the lower-leg placed in the custom-made orthosis to standardize the knee position and ankle movement; **(B)** close-up of the ultrasound probe with reflective markers; **(C)** close-up of the foot attached with an insole to the foot plate of the orthotic. A 6 DoF hand held load-cell was used to measure net ankle joint torque during passive rotation. Two clusters of reflective markers on the tibia and footplate were tracked with motion analysis and used to calculate the foot-plate angle in 3D. The ultrasound probe was placed proximal to the muscle tendon junction, and the position and orientation of the image were defined by motion analysis by means of a cluster of reflective markers attached to the probe. Surface electromyography was collected throughout the experiments from the medial gastrocnemius.

All measurements were performed by the same trained assessor. In children with CP, assessments were carried out on the most affected calf muscle, defined by the highest clinical spasticity grade [Modified Ashworth Scale (20) and Modified Tardieu Scale (21)]. In TDC and in children with CP who were equally affected in both legs, the left leg was selected. Subject body mass and height were measured. Children lay prone on an examination table with the examined limb placed in a custom-made orthotic which allowed knee placement at 20 degrees and free sagittal plane movement at the ankle (Figure 1). The upper leg and pelvis were fixed to the table using straps and the tibia was supported on an inclined cushion with the ankle over the edge of the table. The foot was secured to a rigid footplate with the help of an adjustable insole that ensured heel contact with the footplate during ankle rotation. A six DoF force/torque load-cell (ATI mini45: Industrial Automation) attached to the footplate was used to rotate the ankle joint and measure forces and torques at 200 Hz. The point of contact of the load-cell with the footplate could be adjusted according to the foot size. The moment arms between the lateral malleolus and the point of application of the load-cell were measured using a tape measure.

The angle of the footplate relative to the tibia was recorded using clusters of reflective markers attached to the tibia and footplate tracked by 3D motion analysis cameras (Optitrack NaturalPoint, USA) at a sample rate of 120 Hz. Ankle joint calibration was carried out by pointing to anatomical landmarks on the ankle joint, thereby expressing their relative position to the cluster markers (22).

A B-mode ultrasound (US) scanner (Telemed EchoBlaster128, Vilnius, Lithuania) with a 59 mm linear transducer was fitted with a cluster of reflective markers (Figure 1B) and used to locate and mark the most superficial point of the medial femoral condyle, the medial and lateral borders of the MG muscle and the MG muscle-tendon junction (MTJ). The MG's mid-longitudinal plane was marked from MG origin, through the muscle belly to the MTJ (23).

sEMG electrode location was determined using US on the muscle belly of the MG with an inter-electrode distance of 2 cm (24) (Zerowire, Cometa, Milan, IT). sEMG was sampled at 2,000 Hz. Three repetitions of isometric plantarflexion maximal voluntary contraction (MVC) were performed in prone for normalization purposes. EMG and dynamometry were simultaneously collected via a custom-built modular measurement system (compactRIO, National Instruments, Belgium). Synchronization was achieved by sending a square 5 V signal from the US machine to the motion capture system and to the modular measurement system.

#### Muscle and tendon lengthening during passive motion

A reflective marker was placed over the distal insertion of the Achilles tendon on the most superficial part of the posterior calcaneal tuberosity. Using a custom-made holder, the instrumented US probe was fixed over the MTJ, along the mid-longitudinal plane, and secured to the orthotic to prevent it from moving. Muscle activity, US images, probe orientation, position of the calcaneus marker, ankle torque and ankle kinematics were simultaneously recorded during passive ankle rotations manually applied across the full range of motion (ROM). Three passive rotations were performed, first at slow velocity (5 s to complete full ROM) and then as fast as possible (1 s). Between repeated rotations, there was at least a 7 s rest interval. US images were collected at 30 Hz during slow trials (9) and at 60 Hz during fast trials (11). A close-up video camera was used to retrospectively check heel contact with the footplate during passive rotations.

#### Data reduction

Visual scanning of the quality of the acquired data was performed in custom-made Matlab (R2015a) software. Poor sEMG signal quality was defined as obvious movement artifacts, or a high signal-to-noise ratio. Poor movement performance was defined when the foot was rotated more than 10° outside the sagittal plane or when the heel lost contact with the footplate. Poor imaging was defined when the probe moved 10° outside the movement plane or when contact with the skin was lost. Measurements with incomplete data sets were excluded.

### Data analysis

#### Muscle lengthening parameters

The position of the MTJ in the collected US images was defined as the most distal insertion of the muscle into the tendon. This point, defined in a 2D image, was confirmed to be a representation of the middle of the MTJ, when comparing its location to the location identified in a 3D reconstruction (25). The MTJ was manually tracked in consecutive US images using custom software (26) in Python (2.7). During each slow rotation, the position of the MTJ was defined every 3 frames (on average in 25 images) and during fast rotations, in every frame (on average 60 images). The reliability of such manual tracking of the MTJ has been found to be satisfactory (25). Muscle and tendon lengths were defined as the linear distance between the medial femoral condyle and the MTJ, and between the MTJ and the calcaneus marker, respectively. Muscle tendon unit (MTU) length was defined as the summation of muscle and tendon length. At the start of the motion, the joint was in end-range plantarflexion and all lengths were equated to zero. MTU and muscle lengthening from end-range plantarflexion to end-range dorsiflexion were calculated during slow and fast trials, and expressed in mm. Muscle lengthening was additionally expressed as a percentage of MTU lengthening. Maximum muscle lengthening velocity (MV_MAX_) was calculated as the first derivative of muscle lengthening.

#### Stretch reflex parameters

Data analysis and parameter calculation was carried out with custom software in Matlab. Raw sEMG signals were filtered with a 6th-order zero-phase Butterworth bandpass filter from 20 to 500 Hz. The root mean square (RMS) envelope of the sEMG signal was defined using a low-pass 30 Hz 6th order zero-phase Butterworth filter on the squared signal. Joint angle and angular velocity were calculated from the marker trajectories and ankle calibration. The net ankle joint moment was calculated from the exerted moments and forces on the load-cell, the external moment arms, and the predicted torque caused by gravity on the orthotic (27). All kinematic and kinetic variables were filtered using a 2nd order Butterworth filter with a 6 Hz cut-off frequency.

Ankle flexion-extension ROM and maximum flexion-extension angular velocity (ω_MAX_) were extracted from slow and fast trials. The hyper-activation (EMG gain) of the stretch reflex with increases in joint position and angular velocity was investigated in two EMG parameters following a previously validated approach (7). First, position-dependent increase in the EMG gain during slow trials (EMG_slow_) was calculated as the average RMS-EMG between 10 and 90% of the ROM according to our previous research (7). Second, the time interval in which EMG was on (EMG-onset) during trials was automatically defined according to Staude and Wolf (28). When automatic onset detection failed, a manual correction was applied by visualizing the RMS-EMG signal and, using the ginput function in Matlab, indicating the point at which the signal was visually determined to significantly differ from baseline. To investigate velocity-dependent increase in EMG gain, during fast trials, average RMS-EMG was calculated during EMG onset (EMG_fast_). In trials with no EMG-onset, EMG_fast_ was calculated in an interval 100 ms before to 100 ms after ω_MAX._ All EMG gain values were expressed as a percentage of the peak RMS-EMG during MVC.

When EMG-onset was detected, the latency (in milliseconds) between ω_MAX_ and EMG-onset and between MV_MAX_ and EMG-onset were calculated. Second, the SRT was expressed in terms of the joint angular (ω_SRT_) and muscle lengthening (MV_SRT_) velocities measured 30 ms prior to EMG-onset, which represents the length of the short-latency stretch reflex loop as reported in literature (29).

Work during slow and fast passive rotations was defined as the average area underneath the torque-angle graph from 10% until 90% ROM (27). For the CP group only, during fast rotations, the catch angle, expressed as a percentage of the available ROM was defined as the timing of the first minimum value of the power after the maximum in a power-time curve, according to our previous research (30). Finally, the MG muscle length corresponding to the catch angle was expressed as a percentage of the maximum muscle length. More explanation on these parameters can be found in previous literature (7, 27, 30).

### Statistical analysis

Parameters had a non-normal distribution (Shapiro–Wilk test) and were therefore analyzed with non-parametric statistics. Comparisons of muscle lengthening during slow stretches and of stretch reflex parameters between CP and TDC have been previously reported (10, 27). Therefore, here we only described how parameters changed between slow and fast rotations within each group and compared these change values between groups using Mann-Whitney U tests. Then, within the CP group, Spearman ranks correlation coefficients were calculated between muscle lengthening and stretch reflex parameters at each velocity and, using values averaged over available rotations per subject, between slow and fast velocity. Correlation coefficients were interpreted as poor (<0.2), fair (0.21–0.4), moderate (0.41–0.6), good (0.61–0.8), and very good (>0.8) following the guidelines by Altman (31). Significance of Mann-Whitney U tests was set at *p* < 0.05. For the correlation analyses, a Bonferroni correction was applied resulting in a significance level of *p* < 0.001.

## Results

Of the 38 children who participated in the study, 15 children with CP and 16 TDC had full data sets and were further analyzed (Table 1). Data were excluded from further analysis in two TDC due to incorrect synchronization of signals, in one child with CP due to poor US image quality, in one child with CP due to missing a technical cluster during calibration file, and in two children with CP and 1 TD child due to artifacts in the EMG signal. Patient characteristics (Table 1) were not significantly different between groups.

**Table 1:** Subject characteristics in children with cerebral palsy (CP) and typically developing children (TDC).

**Subject characteristics**	**CP (*n* = 15)**	**TDC (*n* = 16)**
Average (SD) age (years)	11.3 (3.1)	10.3 (4.0)
Male/female (n)	11/4	7/9
Average (SD) Height (cm)	141 (21.3)	138.1 (19.1)
Average (SD) Mass (kg)	36 (18)	35 (15)
Average (SD) tibia length (mm)	339.7 (54.3)	329.4 (52.7)
GMFCS (I-II) (n)	9 I, 6 II	n/a
Involvement level (n)	8 bilateral, 7 unilateral	n/a
Modified Ashworth Score (20) (*n* = 7) and Average Modified Tardieu (21) (*n* = 8)^*^	MAS: 1.5 (*n* = 6); 3 (*n* = 1) Tardieu: 2 (*n* = 5); 3 (*n* = 3)	n/an/a

*GMFCS: gross motor functional classification scale; MAS: modified Ashworth Scale*.

* *Tardieu scores from children recruited at University Hospital of Leuven. MAS from children recruited at Alder Hey Children's Hospital*.

During fast rotations, EMG onsets were automatically detected in 13 subjects of the CP group. In 2 subjects from the CP group and in 2 TDC, automatic detection of EMG onset failed since EMG gain was relatively low and onset was of short duration. In these cases, EMG onset was manually defined.

Table 2 shows median (and IQR) values of muscle lengthening and stretch reflex parameters extracted from slow and fast rotations in CP and TDC. Maximum angular and tissue lengthening velocities were about 30 times higher during fast rotations than slow rotations in both CP and TDC. In CP, this translated to average EMG responses that were, on average, 26 times higher during fast than slow rotations, resulting in higher work values. Higher values of EMG and work in fast vs. slow rotations were also found in TDC, but the increases in EMG and in work values between slow and fast rotations were significantly lower than those of the CP group (Table 2). In both groups, the MTU lengthened by the same amount in slow and fast trials, resulting in a similar ROM. During fast trials, muscle lengthening showed a lower contribution to total MTU lengthening than during slow trials and this tissue behavior was similar in both groups.

**Table 2:** Median (and IQR) of all outcome parameters during sow and fast ankle rotations in children with cerebral palsy (CP) and typically developing children (TDC).

		**CP**		**TDC**		
	**Parameters**	**Slow**	**Fast**	**Slow**	**Fast**	*p*-value^*^
**Joint**	ROM (deg)	40.0 (26.6)	51.9 (19.6)	61.4 (20.7)	66.7 (14.7)	0.45
	ω_MAX_ (deg/s)	12.7 (4.2)	359.5 (178.3)	15.1 (9.8)	464.9 (168.3)	0.04
**Muscle**	Muscle lengthening (mm)	18.3 (9.6)	17.4 (8.8)	26.5 (6.9)	29.2 (11.3)	0.83
	Muscle lengthening (%MTU)	48.6 (9.3)	41.3 (19.8)	61.8 (11.9)	54.4 (12.3)	0.26
	MV_MAX_ (mm/s)	7.6 (2.4)	285.1 (224.8)	9.5 (5.3)	255.8 (106.5)	0.77
**Stretch reflex**	EMG_slow_ (%MVC)	1.3 (2.4)	Na	0.003 (0.2)	Na	Na
	EMG_fast_ (%MVC)	Na	8.1 (9.9)	Na	0.5 (1.0)	< 0.01^†^
	Work (J)	1.0 (0.4)	4.5 (1.6)	1.0 (0.5)	2.9 (1.7)	< 0.01^†^
	Catch angle (%ROM)	Na	82.2 (13.6)	Na	Na	Na
	Catch muscle length (% max MTU)	Na	71.7 (21.5)	Na	Na	Na
	Latency time ω_MAX_ and EMG onset (ms)	Na	−2.5 (72.1)	Na	Na	Na
	Latency time MV_MAX_ and EMG onset (ms)	Na	40.0 (54.2)	Na	Na	Na
	ω_SRT_ (deg/s)	Na	212.1 (154.6)	Na	Na	Na
	MV_SRT_ (mm)	Na	134.0 (186.5)	Na	Na	Na

*MV_MAX_, maximum muscle lengthening velocity; TV_MAX_, maximum tendon lengthening velocity; ω_MAX_, maximum angular velocity; MTU, muscle tendon unit length; ROM, range of motion; EMG_slow_, average electromyography during slow rotation; EMG_fast_, average electromyography during fast rotation; MVC, maximum voluntary contraction; ω_SRT_ angular velocity at the stretch reflex threshold; MV_SRT_ muscle lengthening velocity at the stretch reflex threshold Na, not applicable*.

* *comparison of the change between fast and slow between CP and TDC*.

† *comparison of the change between EMGslow and EMGfast between CP and TDC*.

**Figure 2:**
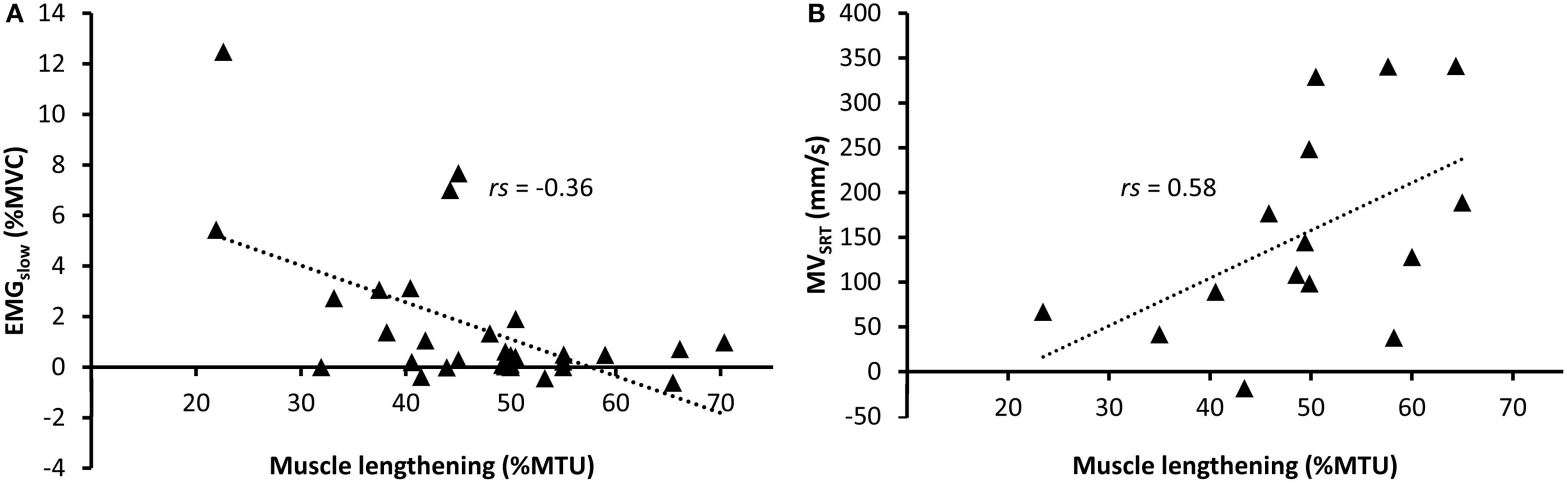
Relative muscle lengthening during slow rotations in children with cerebral palsy vs. **(A)** normalized RMS-EMG during slow rotations and **(B)** average MV_SRT_ during fast rotations. A regression line is shown for significant relationships.

In the CP group, correlation values between muscle lengthening and stretch reflex parameters ranged from poor to excellent (Table 3). During slow rotations, fair negative correlation values were found between EMG_slow_ and absolute (mm) and relative (%MTU) muscle lengthening (*rs* = −0.34, *p* = 0.054; *rs* = −0.36, *p* = 0.042, respectively) indicating higher amount of position-dependent EMG gain in those muscles that were unable to lengthen (Figure 2A). However, none of these results reached significance after the Bonferroni correction of the *p*-value.

**Table 3:** Spearman rank correlation coefficients (and *p*-values) between parameters collected during slow and fast rotations in the cerebral palsy group.

			**Latency time ω_MAX_ and EMG-onset (ms)**	**ω_MAX_ (deg/s)**	**ω_SRT_ (deg/s)**	**Catch angle (%ROM)**	**Latency time MV_MAX_ and EMG-onset (ms)**	**MV_MAX_ (mm/s)**	**MV_SRT_ (mm/s)**	**Catch muscle length (% max muscle length)**	**EMG_fast_**	**Muscle lengthening slow rotation (mm)**	**Muscle lengthening slow rotation (%MTU)**	**EMG_slow_**
**Joint**	**Fast**	Latency time ω_MAX_ and EMG-onset (ms)		−0.097	0.300	0.024	0.471	0.226	−0.237	0.538	−0.199	0.459	−0.015	0.189
		ω_MAX_ (deg/s)	−0.097 (0.562)		0.247	0.039	−0.051	0.313	0.188	−0.188	0.239	0.261	−0.025	−0.402
		ω_SRT_ (deg/s)	0.300 (0.067)	0.247 (0.136)		0.007	−0.013	0.558	0.320	0.083	0.235	0.768	0.254	0.374
		Catch angle (%ROM)	0.024 (0.893)	0.039 (0.830)	0.007 (0.969)		0.368	−0.136	−0.307	0.356	−0.173	−00.549	−0.187	0.023
**Muscle and stretch reflex**	**Fast**	Latency time MV_MAX_ and EMG-onset (ms)	0.471 (0.003)	−0.051 (0.761)	−0.013 (0.940)	0.368 (0.035)		−0.261	−0.660	0.632	−0.490	−0.310	−0.473	0.041
		MV_MAX_ (mm/s)	0.226 (0.172)	0.313 (0.056)	0.558^*^ (< 0.001)	−0.136 (0.449)	−0.261 (0.113)		0.616	−0.047	0.031	0.857	0.204	−0.430
		MV_SRT_ (mm/s)	−0.237 (0.152)	0.188 (0.258)	0.320 (0.050)	−0.307 (0.082)	−0.660^*^ (< 0.001)	0.616^*^ (< 0.001)		−0.448	0.400	0.696	0.575	−0.198
		Catch muscle length (% max muscle length)	0.538^*^ (0.001)	−0.188 (0.279)	0.083 (0.637)	0.356 (0.042)	0.632^*^ (< 0.001)	−0.047 (0.788)	−0.448 (0.007)		−0.503	−0.147	−0.658	0.019
		EMG_fast_	−0.199 (0.252)	0.239 (0.166)	0.235 (0.174)	−0.173 (0.337)	−0.490 ^(0.003)^	0.031 (0.862)	0.400 (0.017)	−0.503 (0.002)		0.033	0.446	0.879
	**Slow**	Muscle lengthening slow rotation (mm)	0.459 (0.003)	0.261 (0.053)	0.768^*^ (< 0.001)	−0.549 (0.029)	−0.310 (0.067)	0.857^*^ (< 0.001)	0.696^*^ (< 0.001)	−0.147 (0.959)	0.033 (0.876)		0.325	−0.421
		Muscle lengthening slow rotation (%MTU)	−0.015 (0.079)	−0.025 (0.809)	0.254 (0.074)	−0.187 (0.446)	−0.473 (0.003)	0.204 (0.030)	0.575^*^ (0.001)	−0.658^*^ (< 0.001)	0.446 (0.042)	0.325 (0.056)		−0.446
		EMG_slow_ (%MVC)	0.189 (0.326)	−0.402 (0.031)	0.374 (0.027)	0.023 (0.908)	0.041 (0.814)	−0.430 (0.020)	−0.198 (0.302)	0.019 (0.926)	0.879^*^ (< 0.001)	−0.338 (0.054)	−0.356 (0.042)	

*ω_MAX_, maximum angular velocity; MV_MAX_, maximum muscle lengthening velocity; EMG_fast_ average electromyography during fast stretches; ω_SRT_ angular velocity at the stretch reflex threshold; MV_SRT_ muscle lengthening velocity at the stretch reflex threshold. Correlation coefficients (31): < 0.2, poor (red); 0.21–0.4, fair (orange); 0.41–0.6, moderate (light green), 0.61-0.8, good (dark green)*.

* *p < 0.001*.

During fast rotations, there was a fair positive correlation of EMG_fast_ with MV_SRT_ (*rs* = 0.40, *p* = 0.017), and a moderate negative correlation with catch muscle length (*rs* = −0.50, *p* = 0.002). There were moderate to good positive correlations between absolute and relative muscle lengthening during slow rotation and MV_SRT_ and MV_MAX_ during fast rotations (Figure 2B). Relative muscle lengthening during slow rotation also had a moderate, but non-significant correlation with EMG_fast_ (*rs* = 0.45, *p* = 0.042), and a good, negative correlation with catch muscle length during fast rotation (*rs* = −0.66, *p* < 0.001).

## Discussion

By providing detailed experimental data on the passive lengthening behavior of muscle and tendon tissue during slow and fast passive ankle rotations, this study innovatively showed that muscle lengthening and stretch reflex hyperactivity in medial gastrocnemius muscles of children with CP is highly variable and that the two do not necessarily co-exist.

The individual forces exhibited on the tissues cannot be assessed *in vivo* and therefore direct quantification of the stiffness of muscle or tendon tissue cannot be defined by means of conventional B-mode ultrasound. However, studying the relative lengthening contribution of the muscle and tendon to the lengthening of the muscle tendon unit, allowed us to make inferences about the muscle's relative tensile behavior during passive ankle rotation. As such, we found that muscles with relatively less muscle lengthening during slow passive ankle rotation showed lower muscle lengthening velocities during fast rotation. On the other hand, muscles with high relative lengthening during slow rotations reached a higher muscle lengthening velocity during fast rotation and were subsequently found to have the largest velocity-dependent stretch reflex responses.

A previous investigation on the same data has shown increased relative tendon lengthening during slow passive MG stretch in children with CP compared to TDC (10). The current study investigated how these findings translate to fast passive stretch of the MG. It was found that, in both TDC and CP, the tendon contributed more toward MTU lengthening during fast stretch than during slow stretch. In the CP group, this additional lengthening from the tendon may be explained by the higher RMS-EMG measured during fast stretch resulting in increased muscle stiffness. However, increased stretch reflex activity cannot fully explain the increased tendon lengthening observed during fast stretch in the TDC. This suggests a velocity-dependent interaction between muscle and tendon during angular rotation in the healthy as well as in the pathological condition. Since there is little evidence of the effect of viscosity on tissue lengthening (32), a possible mechanical stiffening of the muscle may explain this response (33). Lack of a significant difference in velocity-dependent muscle/tendon lengthening in CP vs. TDC questions the pathological role of increased stretch reflexes. On the other hand, this lack of a significant difference may also be explained by a higher angular velocity achieved during fast passive ankle rotation in TDC and likely reflects a larger available ROM in TDC (27).

**Figure 3:**
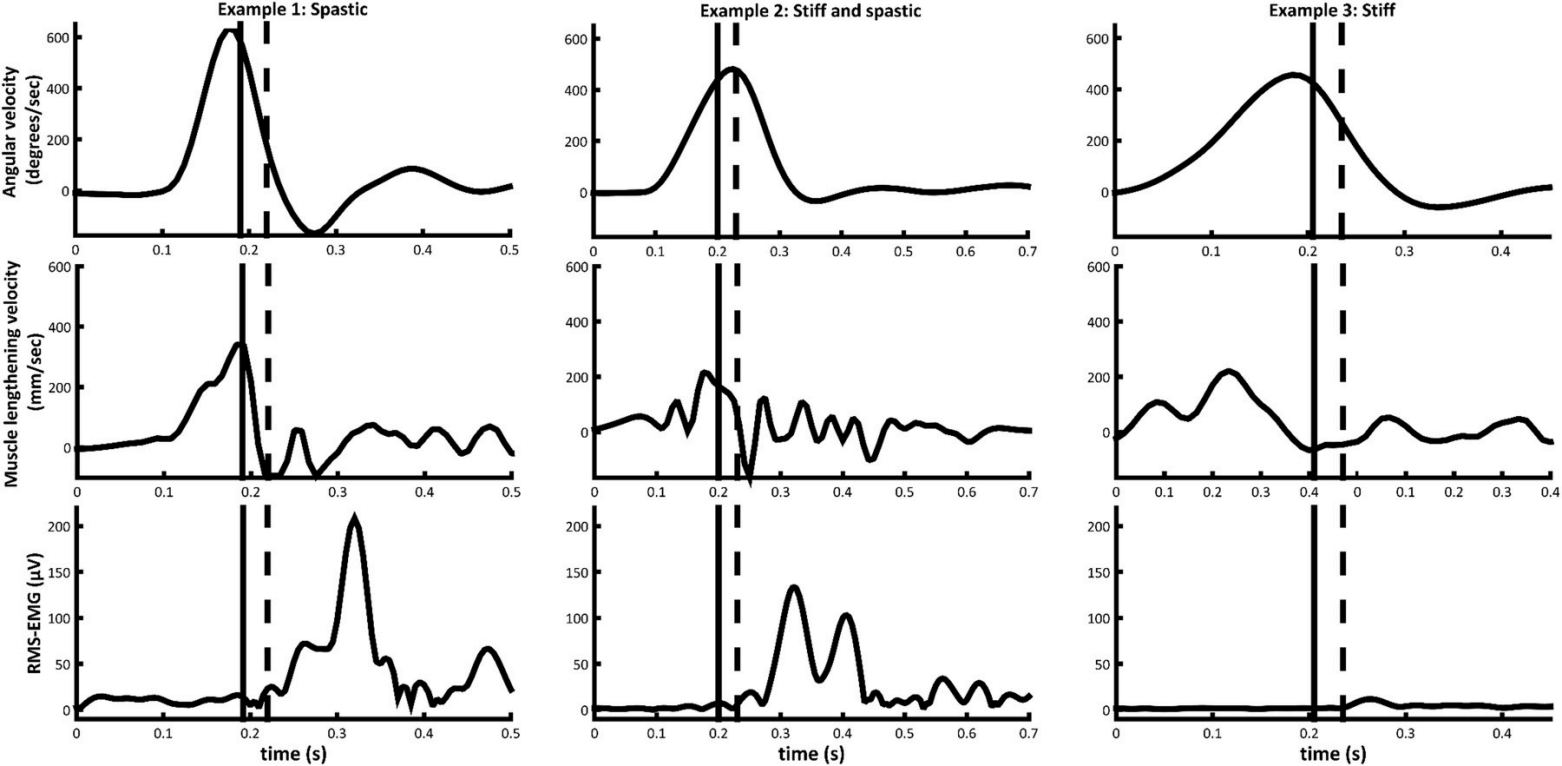
Angular velocity, medial gastrocnemius muscle lengthening velocity, and medial gastrocnemius RMS-EMG during fast passive rotation examples of muscles from three different subjects in the CP group. Timing of EMG-onset (black dashed line) and the stretch reflex threshold 30 ms prior to EMG-onset (black vertical line), are indicated.

The average EMG-onset during fast stretch occurred around 58 ± 43 ms after maximum muscle lengthening velocity. This duration is slightly longer and more variable than the latency time of the short latency reflex (30 ms), described to be involved in the pathophysiology of hyperactive stretch reflexes (34) and also recorded during gait in the MG of children with CP (16). However, after excluding muscles showing an EMG response during fast rotation that was less than 10% MVC (*n* = 4), the latency time decreased to 44 ± 28 ms. Further exploration showed that when only taking muscles (*n* = 4) into account that had muscle lengthening that was greater than the tendon lengthening during slow rotation, the average latency time was 30 ± 18 ms. Therefore, these exploratory findings suggest that a pure velocity-dependent stretch reflex hyperactivity occurs only when the muscle is able to lengthen fast enough. In other muscles, decreased relative muscle lengthening may prevent a large stretch reflex response. Figure 3 shows signals obtained from three individual muscles and highlights the variability in responses.

Neither MV_SRT_ nor MV_MAX_ were in synch with the ω_SRT_ or ω_MAX_, indicating that joint angular velocity was not representative of MG muscle lengthening velocity. This discrepancy could be explained by differences in Achilles tendon moment arm length (35) or in muscle/tendon lengthening properties between subjects. In addition, while ω_SRT_ showed no correlation with parameters of stretch reflex hyperactivity, fair correlations between MV_SRT_ and parameters of stretch reflex hyperactivity indicate that MV_SRT_ might be predictive of EMG gain during fast rotation and an earlier catch. Another indication of the discrepancy between joint angular velocity and muscle lengthening velocity was seen in the latency time between EMG-onset and MV_MAX_ and ω_MAX_. While MV_MAX_ was closely associated with EMG-onset, ω_MAX_ often occurred after EMG-onset. This can be explained by the joint continuing to accelerate in the latency period between the muscle reaching the velocity necessary to elicit a stretch reflex (MV_SRT_) and the muscle force decelerating the joint. Such disparity between angular velocity and muscle lengthening velocity may indicate that the tendon plays a compensatory role in dictating joint behavior. In other words, in stiffer muscles, the tendon is likely to lengthen relatively more to achieve angular rotation. As a result, the muscle does not reach high lengthening velocities. This has some important clinical implications, as it is important to consider that any assessment carried out at a joint level does not reflect the underlying muscle and tendon interactions consistently across all children. For example, clinical decision making based on an assessment of the passive ROM as traditionally performed in a clinical exam, may result in a misdiagnosis of the underlying muscle length. Similarly, gait analysis data reporting only the joint kinematics, and not the underlying muscle-tendon interactions, may be misleading (11, 12). Further, the effectiveness of treatment such as serial casting and stretching that aim to lengthen the muscle by applying stimulus to the joint could be questioned.

During fast rotations, there was a large variability in the magnitude as well as in the timing of muscle activation response, even when the latter was expressed in terms of muscle lengthening instead of angular velocity. Muscles that were found to lengthen less during slow ankle rotation, either showed very little muscle activation response during fast rotation, or, as in the majority of muscles, showed responses that occurred at latencies of more than 30 ms after the maximum lengthening velocity was reached, indicating a response that was not purely velocity-dependent. The relationship between stretch reflex hyperactivity and reduced muscle lengthening is not clear, and the common belief that spasticity, as defined by Lance (2), contributes to the development of stiffer muscle (4) could not be corroborated in this study. Therefore, early aggressive treatment of stretch reflex hyperactivity which has become usual care to delay and reduce the need for orthopedic surgery (5), may not be considered beneficial in all cases.

It has been suggested that increased muscle stiffness, as observed in case of contractures, increases the spindle stimulation from, and its response to, a given amount of lengthening force (8). This would imply that stiffer muscles show greater stretch reflex response to muscle lengthening. Contrary to this, we found that velocity-dependent stretch reflex response was highest in those muscles that were able to achieve a high lengthening velocity. However, similar to previous literature (7, 36–39), we found length-, rather than velocity-dependent muscle activation during slow passive rotation in subjects with CP (EMG_slow_). Although not significant, there was a fair association between low muscle lengthening during slow rotation and higher levels of this type of activation (EMG_slow_). Some authors have used the term *spastic dystonia* to describe a muscle that is over responsive to the degree of a tonic stretch, rather than to its velocity (8). In their research, Dietz and Berger refer to this phenomenon as a “pseudo stretch reflex,” suggesting that its development allows for better gait stability in children with CP (16). Our finding that lower MV_SRT_ values are associated with muscles that lengthened relatively less than their tendon supports this hypothesis. However, we also established the presence of muscles with reduced lengthening that showed very limited muscle response to slow or fast rotation. In such cases, muscle stiffness may actually be considered as a protective mechanism against spasticity. This may question treatments that aim to reduce stiffness in the calf muscles. Does reducing muscle stiffness encourage increased muscle lengthening velocity during angular rotation, and consequently increase the stretch reflex response?

In general, given the large variability in the amount of muscle lengthening and hyperactive stretch reflex in the subject sample included in this study, it is clear that quantification of these per individual muscle is of paramount importance to improve our insight into the underlying mechanisms of increased resistance against stretch and may eventually lead to improved patient-specific treatment. While the measurements described in this study are too complex to carry out in regular clinical practice, future research should identify those markers that associate with our findings but that are easier to collect in the clinic [for example, assessment of static muscle morphology using US (40)]. Furthermore, the current study indicates that differences in material properties of the muscle tendon unit cannot fully explain the variability in the stretch reflex. Other regulatory pathways of muscle spindle sensitivity should therefore be considered. For example, one study reported lower medial gastrocnemius growth rate in young children with unilateral compared to bilateral involvement (41), yet our research group could not confirm this (article submitted). While the current study was too small to allow for any sub-group analyses, future studies could investigate whether level of involvement, age, or markers in the original brain insult (for example using MRI) are related to stretch reflexes.

Limitations of the current study need to be mentioned. The study had a small subject sample. Upon *post-hoc* analysis, some of the statistical tests correlating reflex and muscle lengthening outcome parameters may not have been sufficiently powered. Therefore, the results of this study should be used to guide the choice of outcome measures and required sample sizes for future work. Other plantarflexors that insert into the Achilles tendon influence the force in the tendon and the movement of the MG MTJ. However, in CP, we expect the MG to be the most impaired in terms of stretch reflex activation and muscle shortening (3). Since the experiments were complex, we only investigated the most affected leg in children with CP while a comparison of the affected to unaffected legs would have been interesting to study, especially in subjects with unilateral involvement. We used a 2D imaging technique to visualize and track the movement of 3D structures. While this is an inherent limitation of dynamic US imaging, our results of limited muscle belly lengthening in children with CP are in agreement with previous literature (42). Furthermore, data from our research group involving a comparison of 2D to 3D US images collected across the ankle ROM in children with CP, indicated that this approach is valid for the purpose of the current investigation (25). During fast rotation acquisitions we sampled US images at 60 fps which means that tissue lengthening velocities at 30 ms prior to EMG-onset were defined in only 2 frames. This may also explain why some latencies were longer than expected. RMS-EMG was normalized to the muscles' MVC, which is challenging to collect in children with CP (43). Yet, alternative normalization methods, e.g., normalization to muscle activity during a known force, tended to produce more variable results while others were considered too invasive for use in children e.g., M-wave excitation. Moreover, the MVC as applied here has previously shown sufficiently reliable (44) and the current study also included variables related to the timing of EMG-onset that prevented the need for normalization. This investigation was carried out during passive joint rotation. The assumption that stretch reflexes as assessed during passive rotation occur when the muscle is activated voluntarily or in an upright posture cannot be made. Although discerning the occurrence of stretch reflexes during active muscle lengthening is challenging, future research should attempt to investigate the impact of stiffness and stretch reflexes on the performance of functional activities. It is possible that muscles that were found to be less stiff were stretched at higher velocities by the examiner thus eliciting a stretch reflex response. Similarly, ankles from the TDC were rotated at a faster velocity compared to CP. Passive rotation imposed by a robotic device would have allowed for more controlled ankle angle manipulation. However, the velocity profile achieved with a manual rotation better mimics the rotation pattern of the ankle during gait (45) and is found to more often elicit stretch reflexes (46). Finally, due to foot deformations in the CP group, we cannot exclude the possibility that the axis of the orthotic was not always perfectly aligned with the ankle axis. However, since a similar method was used to secure the foot to the orthosis in all measured subjects, we do not expect this to have influenced our results.

## Conclusions

We established different patterns of tensile muscle behavior in the tested children with CP: 1. muscles with high muscle lengthening allowing high muscle lengthening velocities that elicit a stretch reflex; 2. muscles with little lengthening, preventing high muscle lengthening velocities, that exhibit length-dependent muscle activation at low stretch velocities; and 3. muscles with little lengthening and little to no reflex activation at either slow or fast rotation. Given this large variability between children with CP, treatments directed at the medial gastrocnemius that aim to decrease ankle joint hyper-resistance should ideally be based on quantification of the amount of stretch reflex hyperactivity and stiffness at the isolated muscle-tendon tissue level, rather than gross measurement of passive resistance at whole joint level. In addition, muscle and tendon lengthening properties alone could not fully explain the variability in stretch reflexes, indicating that also other factors should be investigated.

## Data availability statement

The processed data supporting the conclusions of this manuscript will be made available by the authors, without undue reservation, to any qualified researcher.

## Author contributions

LB and BK are shared first authors. LB, BK, TO, KD, GB, and CM conceptualization. LB, BK, and FC data curation. LB and BK formal analysis. LB, KD, and TO funding acquisition. LB, BK, KD, TO, GB, CM, FC, S-HS, and GH investigation. LB, FC, and S-HS methodology. LB, BK, TO, and GH project administration. TO, KD, GH, AB, and GM resources. FC, BK, and LB software. KD, CM, GB, and TO supervision. FC, LB, and BK validation and visualization. LB and BK writing—original draft. LB, BK, KD, TO, GB, CM, FC, S-HS, GH, AB, and GM writing—review and editing.

### Conflict of interest statement

The authors declare that the research was conducted in the absence of any commercial or financial relationships that could be construed as a potential conflict of interest.

## Abbreviations

CP: Cerebral palsy
DoF: Degrees of freedom
EMG_fast_: Increase in the EMG gain during fast trials
EMG_slow_: Increase in the EMG gain during slow trials
MG: Medial gastrocnemius
MTJ: Muscle tendon junction
MTU: Muscle tendon unit
MVC: Maximum voluntary contraction
MV_MAX_: Maximum muscle lengthening velocity
MV_SRT_: Muscle lengthening velocity measured 30ms prior to EMG-onset
RMS: Root mean square
ROM: Range of motion
sEMG: Surface electromyography
SRT: Stretch reflex threshold
TDC: Typically developing children
US: Ultrasound
ω_MAX_: Maximum ankle angular velocity
ω_SRT_: Ankle joint angular velocity measured 30ms prior to EMG-onset
